# What of the Man?

**Published:** 1919-09-13

**Authors:** 


### WHAT OF THE MAN?

Dr. Mercier's was a complex character. He was born to suffer, may we say unduly, having regard to the extent and character of such suffering and the long period of his life during which he was so afflicted. Naturally he was one of the kindest, most generous, and most helpful of friends. He lived in an atmosphere where friendship was to him probably the greatest and most treasured element. To a friend in difficulty or doubt or illness or trouble of any kind he brought all his powers and faculties to render aid. Nothing was too much trouble; no strain was too great; and no demand too large to satisfy the abundance of real goodness which existed in Mercier's character. Even when ill, if there was something to be done or some specially difficult and technical work to turn out which pressed, Mercier had a wonderful faculty of bringing his mind to the task and giving the strength, in God's mercy, to produce the copy without fail. This was a remarkable power, generously used on many occasions, and one, alas! so rare to meet as to make it a treasure beyond price. "Keep your friendships in repair" was a precept which he took from Dr. Samuel Johnson and adopted in practice as his own, teaching its value and effect on this earth.

No man was more conscious than he of his faults and failings, for he had both, as we all have, and we know, from a somewhat close and intimate acquaintance with the man, that he set himself to eliminate defects of this kind, and succeeded in a remarkable degree. We have had much evidence within the last two years of the goodness of heart, natural affection and tenderness which underlay much of the rough exterior of a great mind. Mercier was a friend of surpassing value to those whom he honoured with his friendship.

One lesson all may learn from Charles Mercier, one of the greatest sufferers of his time, who, in face of the grievous recurrence of agonies and disablements, learnt to master himself and mask his feelings so fully, as to enable him, in the closing months of his life, to produce some excellent work, and to complete, to his great joy, an engagement he had made with the man who first brought to his notice the intensive system of agricultural development by writing and completing an important paper on this subject, recording the progress and high position in fruitfulness which had already resulted from its adoption. This paper he hoped so recently as ten days ago, despite his blindness and infirmities, to present in person to the assembled members of the British Association at their meeting at Bournemouth, commencing Tuesday, September 9. When the last hours came and death proved insistent, among his closing thoughts and acts was the handing of his MS. to his attendant physician, who gave him the promise that he would attend the British Association, explain the cause of Mercier's absence, and read the paper on his behalf. We have not a doubt that Mercier's hope and intention were to attend the meeting, to have his paper read by a thoroughly good reader, to invite discussion, and have the satisfaction of meeting those who might still remain captiously critical and unbelieving in regard to the benefits of the system, which he had done so much to extend. If there were sceptics still, those sceptics must be met and answered. So it was Mercier's intention, we have not a shadow of doubt, to have answered them, as he would have done, with all the vigour he possessed, and so end his days as a valiant warrior, unvanquished and assured. Dryden's words are apposite in this connection, and might well find a place on Charles Mercier's tomb:

Rich the treasure,
Sweet the pleasure,
Sweet the pleasure after pain.

Mercier as a last act sent a line of intimation to his most intimate friend, and passed away peacefully, in the hope of a joyful resurrection.

